# New taxonomic data on the genus *Ypsolopha* Latreille (Lepidoptera, Ypsolophidae) with descriptions of two new species from the Russian Far East

**DOI:** 10.3897/zookeys.289.3905

**Published:** 2013-04-12

**Authors:** Margarita G. Ponomarenko, Yuliya N. Zinchenko

**Affiliations:** 1 Institute of Biology and Soil Science, Far Eastern Branch of Russian Academy of Science, pr. 100–letiya, 159, Vladivostok 690022, Russia; 2 Far Eastern Federal University, Oktyabrskaya str., 27, Vladivostok, 690091, Russia

**Keywords:** Lepidoptera, Ypsolophidae, *Ypsolopha*, new species, new synonymy, first record, Russia, Far East

## Abstract

Two new species of the genus *Ypsolopha* Latreille, 1796 are described from Far East of Russia: *Ypsolopha melanofuscella* **sp. n.** and *Ypsolopha straminella* **sp. n.** Two new synomymies are proposed: *Ypsolopha ulingensis* Yang, 1977, a new junior synonym of *Ypsolopha costibasella* (Caradja, 1939); and *Cerostoma falculella* Erschoff, 1877, a new junior synonym of *Ypsolopha asperella* Linnaeus, 1761. The species *Ypsolopha costibasella* Caradja, 1939, *Ypsolopha nigrofasciata* Yang, 1977 and *Ypsolopha nigrimaculata* Byun et Park, 2001 are recorded from Far East of Russia for the first time. The male genitalia of *Ypsolopha nigrofasciata* are described and illustrated for the first time, diagnostic genital characters are given.

## Introduction

The genus *Ypsolopha* Latreille, 1796, comprises more than 130 species worldwide (Moriuti 1977; Zagulyaev 1981; Agassiz & Friese 1996; Gershenson 1997; Dugdale et al. 1998; Sinev 2008; Zinchenko & Ponomarenko 2008; Pogue 2009; Sohn et al. 2010; with the centre of species diversity in Eastern Asia. In several recent published papers the history of the study of the genus in Asia was reviewed, all earlier published data were summarized, the types of previously described species were revised, new species were described and a checklist of the East Asian species was compiled (Byun & Park 2001, Sohn et al. 2010, Ponomarenko et al. 2011, Sohn 2011). However, in spite of the attention paid to this group in the last two decades, the study of its species diversity is not complete. The present paper continues the recent series of publications on Asian *Ypsolopha*. It is the purpose of this paper to establish new synonymies, specify the distribution of three known species: *Ypsolopha costibasella* Caradja,1939, *Ypsolopha nigrofasciata* Yang, 1977 and *Ypsolopha nigrimaculata*, Byun et Park, 2001, describe the previously unknown male genitalia of *Ypsolopha nigrofasciata*, improve the diagnosis for the latter, including genital characters, and describe two new species: *Ypsolopha melanofuscella* **sp. n.** and *Ypsolopha straminella* **sp. n.** The description of two new species increases the number of East Asian *Ypsolopha* species to 49 (see Sohn et al. 2010).

## Material

The study is based mainly on material collected in the southern part of the Russian Far East and preserved in the Institute of Biology and Soil Science of the Far Eastern Branch of the Russian Academy of Sciences (Vladivostok, Russia). Additional material from the Zoological Institute of the Russian Academy of Sciences (Sankt–Petersburg, Russia) was studied and data on the type specimens kept in Muzeul Naţional de Istorie Naturală “Grigore Antipa”, (Bucharest, Rumania) were included. Depositories of the types and other specimens are indicated at the end of the label data or, in the case of multiple records from the same collection, after the last one.

### Abbreviations of depositories

**IBSS** Institute of Biology and Soil Science, Far Eastern Branch, Russian Academy of Sciences, Vladivostok, Russia;

**MGAB** Muzeul Naţional de Istorie Naturală “Grigore Antipa”, Bucharest, Rumania;

**ZINRAS** Zoological Institute, Russian Academy of Sciences, Sankt–Petersburg, Russia.

The verbatim labels of the type specimens described by A. Caradja and N. Erschoff are cited in quotes, missing figures and names in the old labels are added in square brackets.

## Methods

For genitalia dissection, abdomens of moths were detached and, following maceration in 10% KOH, dissected and examined in glycerol. The membranous parts of the genitalia were stained with Chlorazol Black. The genitalia of both sexes were mounted in slide in Euparal. Genitalia slides were prepared by the first author (MP). and subsequently examined using a stereomicroscope Nikon SMZ–10. Photographs of adults and genitalia were made with digital camera Nikon Coolpix 8700. In the descriptions, the terminology for genitalia follows Klots (1970) after modifications according to Kuznezov & Stekolnikov (2001). For the descriptions of the parts of the phallus we use the following terms: for the phallic sclerotized tube: aedeagus, for the phallotheca: anellus, for the endophallus: vesica with cornuti. Those correspond to accepted morphological terminology (Snodgrass 1935, Kuznetzov & Stekolnikov 2001, Kristensen 2003). The size of genital sclerites is given as relative size of other genital parts. For the purpose of measurements the membrane between segments IX and VIII is defined from the base of the papillae anales to the posterior margin of the ventral sclerotization of segment VIII; the apophysis anterior from its basal bifurcation to the apex; segment VIII from the posterior to the anterior margin of the ventral sclerotization. We composed scientific names of new taxa following Borror (1971) and Kirpicznikov & Zabinkova (1977).

### New nomenclatural and faunistic data

#### Ypsolopha asperella

(Linnaeus, 1761)

http://species-id.net/wiki/Ypsolopha_asperella

Figs 1 , 7, 8

Phalaena (Tinea) asperella Linnaeus, 1761: 369; type locality: Upsala, Sweden.
Cerostoma falculella Erschoff, 1877: 343; type locality: Irkutsk, Russia; **syn. n.**

##### Material examined:

**Type.** Lectotype of *Cerostoma falculella* (here designated): ♂, [Russia], “Irkutsk VII [18]66”; “coll. Erschov”, gen. slide Yps.–29 MP; red rectangular label “Lectotype *Cerostoma falculella* Erschoff, 1877” (ZINRAS).

##### Additional material.

Russia. 1♂, Peterburg [St.–Petersburg] suburb, 21 April [19]06, N. Kuznetcov leg., gen. slide Yps.–39 MP; 1♀, Pskov, 5 October 1907, Chistovsky leg.; 2♀, Irkutsk, coll. Grand Prince N.M. [Romanoff], gen. slide Yps.–32 MP, Yps.–33 MP (ZINRAS); 1♀, Chitinskaya obl., Nizhnii Chasuchei, 2 June 1995, V. Dubatolov, R. Dudko leg., gen. slide Yps.–34; 1♂, Jewish Autonomous Region, Radde, 17 July 2005, M. Ponomarenko leg.; Primorskii krai: 1♀, Sinegorka Mt. foot, 2 August 1999, M. Ponomarenko leg.; 1♂, Ussuriyskii reserve, 10 May 1995, Chistyakov leg.; 2♂, 3♀, 20 km SE Ussuriysk, Gornotaezhnoe, 20 August, 2 October 1994, 28, 29 April 1995, M. Ponomarenko leg., gen. slide Yps.–35 (♂) MP; 2♀, Vladivostok, Botanic garden, 17 April 1996, E. Beljaev leg.; 1♂, Shkotovskii distr., Litovka Mt., 22–25 October 1998; 1♂, Anisimovka, 9 October 1999, gen. slide Yps.–36 MP; 1♂, Chuguevskii distr., 16 km SE Yasnoe, Ussuri riv., “Pobedinskaya Polyana” hole, 2 August 2012, M. Ponomarenko leg.; 1♂, Sakhalin, Yuzhno–Sakhalinsk, 28 August 2010, V. Dubinina leg., gen. slide Yps.–37 MP (IBSS). Belorussia. 1♂, Vitebsk, 3 September 1975, V.I. Piskunov leg., gen. slide Yps.–38 MP (ZINRAS). Ukraine. 1♂, 1♀, Sumskaya obl., Lebedin, 10, 13 August 1968, V.I. Piskunov leg., gen. slide Yps.–30 (♂) MP, Yps.–31(♀) MP (ZINRAS).

##### Distribution.

Europe, Asia Minor, Middle East, Russia (European part, South Ural, Irkutskaya oblast’, Transbaikalia, Amurskaya oblast’, Primorskii krai), Korea and China (Beijing).

##### Remarks.

Our examination of the lectotype of *Cerostoma falculella* and specimens of *Ypsolopha asperella* from Europe, South Siberia and Russian Far East did not show sufficient differences to justify treating them as separate species. It should be noted that in the genitalia of the lectotype of *falculella* (Fig. 7) the ventral margin of the valva is slightly angled as it is in Far Eastern specimens of *Ypsolopha asperella*. Other characters (external and genitalic) can be more or less variable. No specific external and genitalic characters correlated with a particular generation were found nor any characters typical of European or Asian populations. Every generation whether of European or Asian populations contains specimens expressing the full spectrum of character variability. The life cycle of this species is studied insufficiently and it is difficult to ascertain how many generations there are. According to the examined material and published data the adults fly in the spring from March-May (Zagulyaev 1981; Gershenson 1997). Usually spring specimens appear worn with indistinct forewing pattern, suggesting that specimens flying in October overwinter and reappear in early spring. In summer the adults appear in July. Moths flying in spring and summer vary in wing span from 16 mm to 22 mm. The aedeagus in every studied specimen has two more or less recessed apical lobes, 1–3 large square apical denticles and 3–14 small preapical ones (Figs 8a-f). The lectotype of *Cerostoma falculella*, collected in July, has an aedeagus with two large square apical denticles (Fig. 8a), the same number as found in specimens of *Ypsolopha asperella* from Vitebsk (Belorussia) (Fig. 8e) collected in September and Anisimovka (Russian Far East) (Fig. 8f) collected in October. Specimens of *Ypsolopha asperella* from Sankt-Petersburg (Russia, European part) (Fig. 8c) and Gornotaezhnoe (Russian Far East) (Fig. 8b), both collected in April, have a single square apical denticle on the aedeagus whilst in specimens from Sumskaya obl. (Ukraine) collected in August the aedeagus bears three large square apical denticles. Thus, the number of apical denticles on the aedeagus is not correlated with spring and summer generations nor with the geographic distribution, and it is here interpreted as intraspecific variation. Other structures of the male and female genitalia in specimens from different populations are more or less uniform and do not demonstrate distinct differences. Therefore *falculella* Erschoff is considered to be a junior subjective synonym of *asperella* Linnaeus.

**Figures 1–6.**
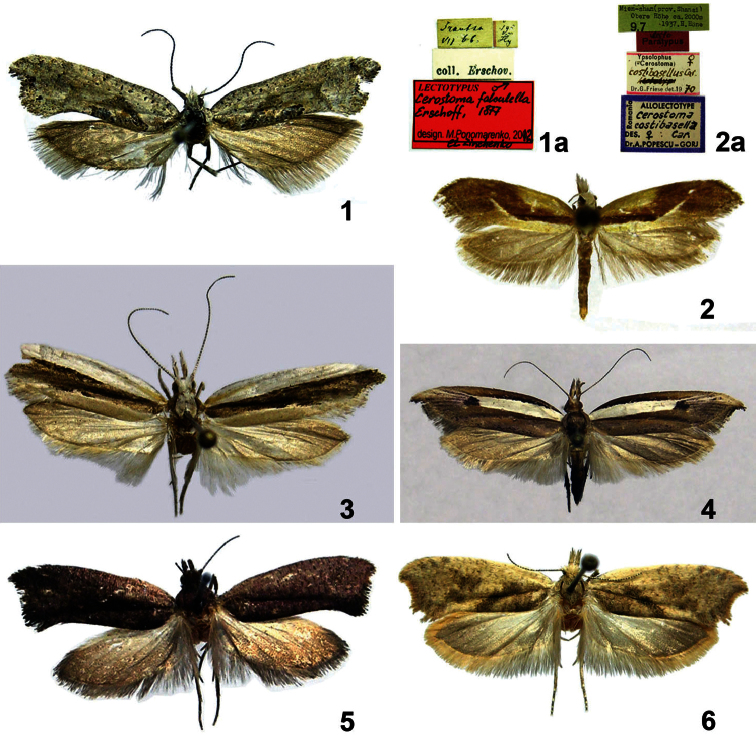
Adults of *Ypsolopha* spp. **1** *Ypsolopha asperella*, lectotype, Russia **1a** ditto, labels **2** *Ypsolopha costibasella*, allolectotype, China (photo by Mr. George Nazareanu, MGAB) **2a** ditto, labels **3** *Ypsolopha nigrofasciata*, Russia **4** *Ypsolopha nigrimaculata*, Russia **5** *Ypsolopha melanofuscella* **sp. n.**, holotype, Russia **6** *Ypsolopha straminella* **sp. n.**, holotype, Russia.

**Figures 7–12.**
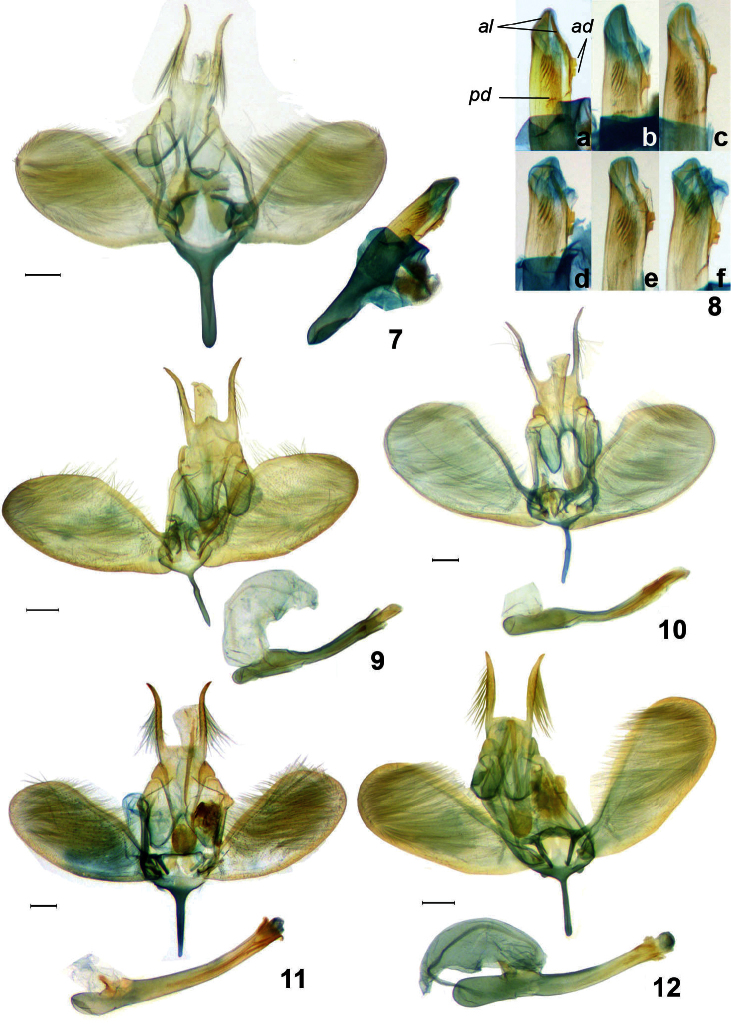
Male genitalia of *Ypsolopha* spp., ventral view. **7** *Ypsolopha asperella*, lectotype, Russia **8 a–f** *Ypsolopha asperella*, distal part of aedeagus: **a** lectotype, Irkutsk, South Siberia **b** specimen from Gornotaezhnoe, Russian Far East **c** specimen from Sankt-Petersburg, European part of Russia **d** specimen from Sakhalin, Russian Far East **e** specimen from Vitebsk, Belorussia **f** specimen from Anisimovka, Russian Far East **9** *Ypsolopha costibasella*, Russia **10** *Ypsolopha nigrofasciata*, Russia **11** *Ypsolopha melanofuscella* **sp. n.**, holotype, Russia **12** *Ypsolopha straminella* **sp. n.**, holotype, Russia. Aedeagus in lateral view. Scale 0,2 mm. ***al*** apical lobes ***ad*** apical denticles ***pd*** preapical denticles.

#### Ypsolopha costibasella

Caradja, 1939

http://species-id.net/wiki/Ypsolopha_costibasella

Figs 2 , 9

Cerostoma costibasella Caradja, 1939: 14; type locality: Mien–Shan, Shanxi, China.
Ypsolophus ulingensis Yang, 1977: 106, figs. 6, 7; type locality: Lingwushan, Hebei, China; **syn. n**.

##### Material examined:

**Type.** Paralectotype of *Cerostoma costibasella*: ♀, [China], “Mien–Shan (prov. Shansi), Obere Höhe ca. 2000 m 9.7.1937. H. Höne”, “allolectotype of *Cerostoma costibasella* Car.” (designated by A. Popescu–Gorj) (MGAB).

##### Additional material.

Russia. Primorskii krai: 1♂, 20 km SE Ussuriysk, Gornotaezhnoe, 43°41'42"N, 132°09'24"E, 21 July 1994, gen. slide Yps.–40 MP; 3♂, 2♀, Chuguevskii distr., 39 km E Yasnoe, Snezhnaya Mt., altitude 1230 m, 43°42'56"N, 134°26'15"E, 31 July–1 August 2012, M. Ponomarenko leg. (IBSS).

##### Distribution.

China (Beijing, Hebei, Shanxi), Russia (Far East, first record).

##### Remarks.

The lectotype of *Cerostoma costibasella* Caradja, which was designated, together with an allolectotype, by Popescu-Gorj (1992), has not been found in MGAB, Bucharest. According to its label the ‘allolectotype’ was collected in the same locality as the lectotype but two days earlier. Certainly the allolectotype is conspecific with the lectotype as indicated by the unique wide lemon yellow costal fascia in the basal half of the forewing. This character was mentioned in the original description by A. Caradja (1939): “ ... ein breiter zitronengelber Kostalstreifen von Basis bis 1/2 ...”. Recently the type of *Ypsolopha ulingensis* Yang was studied and illustrated by Sohn (2011) and found to be identical in wing pattern with *Ypsolopha costibasella*, which was described from the neighbouring province of Shanxi and also from the mountains. An additional series of specimens of both sexes was collected in the Russian Far East on Mt. Snezhnaya. Their wing pattern is identical with that in *Ypsolopha costibasella* and *Ypsolopha ulingensis*, and their male and female genitalia agree with those of the latter illustrated by Sohn (2011). *Ypsolopha ulingensis* Yang is therefore considered to be a junior subjective synonym of *costibasella* Caradja.

#### Ypsolopha nigrofasciata

Yang, 1977

http://species-id.net/wiki/Ypsolopha_nigrofasciata

Figs 3 , 10

Ypsolophus nigrofasciatus Yang, 1977: 105, fig. 5; type locality: Lingwushan Mt., Hebei, China.
Ypsolopha nigrofasciata : Sohn et al. 2010: 24.

##### Material examined.

Russia. Primorskii krai: 2♂, Shkotovskii distr., Berezovyi stream, 6 km S Anisimovka, altitude 450 m, 43°07'28"N, 132°47'44"E, 24 August 2002, M. Ponomarenko leg., gen. slide Yps.–81 MP (IBSS).

##### Diagnosis.

In the male genitalia *Ypsolopha nigrofasciata* Yang resembles *Ypsolopha yasudai* Moriuti, 1977 in the valva bearing a knob at two-thirds of costal margin, a wide rounded excavation between the anterior lobes of the tegumen, medially prominent posterior margin of the uncus and two cornuti of less than one-half the length of the aedeagus.

*Ypsolopha nigrofasciata* is distinguished by the distinctly constricted median plate of the gnathos with dilated proximal part, the distally conical anterior lobes of the tegumen and the straight basal third of the aedeagus. The forewing is characterized by the longitudinal brown median fascia and the white costal and greyish anal areas (Fig. 3). In *Ypsolopha yasudai* the median plate of the gnathos has parallel margins, the anterior lobes of the tegumen are broadly rounded distally and the aedeagus is gently arched.

##### Male genitalia

(Fig. 10). Uncus 0.6 times width of vinculum, with triangular projection on posterior margin; socii long, setose, 2.3 times length of median plate of gnathos, tapering towards pointed apex, slightly sinuous. Gnathos constricted just before dilated part of median plate, median plate 0.4 times width of uncus. Tegumen with rounded emargination between anterior lobes, reaching one-half length of tegumen; anterior lobes distally conical. Valva obovate, rounded distally, widest at distal 1/3, considerably narrowed towards base, with distinct knob on costal margin at 2/3 of valvar length; costa relatively narrow, reaching 1/6 length of valva; saccular area one-half width of costa , reaching apex of valva. Vinculum band–like, arched laterally, narrow saccus with parallel margins, slightly shorter than socii. Aedeagus almost straight in basal third and gently arched at middle; coecum about 1/9 length of aedeagus; two cornuti less than one-half length of aedeagus.

##### Distribution.

China (Hebei),Russia (Far East,first record).

#### Ypsolopha nigrimaculata

Byun et Park, 2001

http://species-id.net/wiki/Ypsolopha_nigrimaculata

Fig. 4

Ypsolopha nigrimaculatus Byun et Park, 2001: 2; type locality: Gyebangsan Mt., Prov. Gangwon, Korea.
Ypsolopha nigrimaculata : Sohn et al. 2010: 24.

##### Material examined:

Russia. Primorskii krai: 2♂, 3♀, Khasanskii distr., 14 km SW Slavyanka, Ryazanovka, 42°47'36"N, 131°15'06"E, 16 August 2010, M. Ponomarenko leg. (IBSS).

##### Distribution.

Russia (Far East,first record), Korea.

##### Remarks.

This species was previously known only from South Korea. A series of five specimens was collected for the first time in the southernmost part of Primorskii krai.

### Descriptions of new species

#### Ypsolopha melanofuscella sp. n.

urn:lsid:zoobank.org:act:5D69836A-E63A-495D-8F48-8E10F7DAD75A

http://species-id.net/wiki/Ypsolopha_melanofuscella

Figs 5 , 11 , 13

##### Type material.

**Holotype**: ♂, Russia, Primorskii krai, Ussuriyskii district, Gornotaezhnoe, 43°41'42"N, 132°09'24"E, 28 August 1994, M. Ponomarenko leg., gen. slide Yps.–59 MP (IBSS); labeled by red label with designation “Holotype”. **Paratypes** (9♂, 1♀): Russia, same locality and collector as in holotype, 20 July 1994, gen. slide Yps.–58 (♂) MP; 29 August 1994, 2 October 1994, gen. slide Yps.–56 (♀) MP; 22 September 1995, gen. slide Yps.–55 (♂) MP; 23 September 1995, gen. slides Yps.–57 (♂) MP, Yps.–60 (♂) MP (IBSS).

##### Diagnosis.

Externally the new species is extremely similar to *Ypsolopha atrobrunnella* Ponomarenko et Sohn, 2011. It can easily be distinguished by the smaller size and the genitalia, especially those of the female, the total length of which (measured from the apex of the papillae anales to the bottom of the corpus bursae) is 1.7 times the length of that in related species. The new species also differs in the male genitalia in the narrower anellus and cornuti with the distal needle-like part 1/6^th^ the total length of the cornutus (in *atrobrunnella* almost one-half length of the cornutus), in the distally (from 1/2 to 4/5) almost parallel dorsal and ventral margins of the valva (in *atrobrunnella* evenly narrowing towards base from dilation at 4/5); in the female genitalia in the absence of a spinose zone in the ductus bursae (in *atrobrunnella* ductus with a broad band of densely set spines), and a signum with the anterior part wider than the posterior (in *atrobrunnella* the band–like signum dilated posteriorly).

##### Description.

**Adult** (Fig. 5).Head covered with appressed elongated narrow dark brown scales with light tips. Antenna filiform; scape dark brown with light grey pecten; each flagellomere dark brown in basal half, white in distal half. Labial palpus porrect and slightly curved upwards, pointed terminally; mainly covered by dark brown scales with white tips; basal segment ventrally white; second segment dorsally and ventrally white, with brown triangular ventral tuft; third segment as long as second, inner surface pale grey. Thorax and tegula dark brown with violet lustre. Foreleg with femur pale grey; tibia greyish brown; each tarsomere dark brown, distally with narrow white ring. Midleg with light grey femur and greyish brown tibia and tarsus. Hindleg with femur and basal two-thirds of tibia light grey, distal third of tibia and tarsus greyish brown; tibial spurs brown dorsally and white ventrally. Forewing length 7.5–8.0 mm (n = 6), wingspan 16.5–17.3 mm (n = 4), sub-trapezoidal, with falcate, obtuse apex and sinuate termen, dark brown with violet lustre; with weakly visible transverse striation; fringe dark brown. Hindwing dark brownish grey, paler towards base; fringe dark grey.

**Male genitalia** (Fig. 11). Uncus slightly narrower than vinculum, with almost straight posterior margin; socii with long setae, 2.6 times length of median plate of gnathos, tapered towards apex, basal 5/6 with parallel margins, distal part diverging. Gnathos with median plate dilated at middle, about one-half uncus width. Tegumen with dorsal triangular median sclerotization narrowing caudally, divided into pair of anteriorly rounded lobes, dorsal notch about 1/3 length of tegumen. Valva obovate, distally rounded, widest at 4/5, margins almost parallel from 1/2 to 4/5, much narrower towards base; costa very narrow, reaching distal third of valva; saccular area equal in width to costa, about 4/5 length of valva. Vinculum band–like, arched laterally, saccus slightly longer than socii, tapered towards apex. Anellus setose, as wide as basal part of valva. Aedeagus almost straight, with cylindrical apex; coecum 1/6 length of aedeagus, slightly inflated in proximal part and wider than distal part of aedeagus; two long cornuti less than one-half length of aedeagus, each with distal needle–like part of 1/6 length of cornutus.

**Figures 13, 14.**
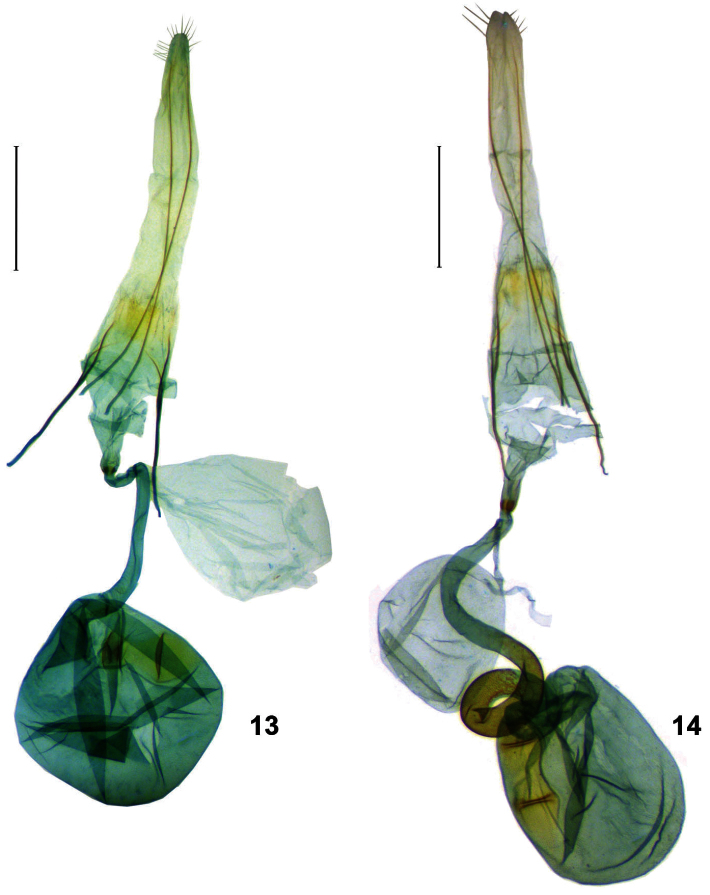
Female genitalia of *Ypsolopha* spp., ventral view. **13** *Ypsolopha melanofuscella* **sp. n.**, paratype, Russia **14** *Ypsolopha straminella* **sp. n.**, paratype, Russia. Scale 0,5 mm.

**Female genitalia** (Fig. 13). Papilla analis semioval, ovipositor telescopic, intersegmental membrane between IX and VIII almost 5.5 times length of VIII. Apophysis posterior slender, almost reaching apex of papilla analis, thickened anteriorly, 3.7 times length of apophysis anterior; apophysis anterior with Y–shaped base, its ventral branch stretching lateroventrally along anterior margin of VIII. Ventral sclerotization of VIII with sinuous anterior margin, in distal 1/3 divided into pair of lobes, bearing long setae on rounded apex. Ostium slightly wider than one-half of segment VIII; antrum cone–shaped, separated from ductus bursae by colliculum as ring–like sclerotization concave inward dorsally, ventral part of it as narrow band and dorsal one 3 times as wider. Ductus bursae tubular, membranous, 1.4 times length of corpus bursae; bulla seminalis almost length of corpus bursae, with ductus seminalis 1/11 length of bulla seminalis, arising from distal 1/16 of ductus bursae. Corpus bursae ovate, membranous; signum band–like, dilated anteriorly, with two transverse ridges.

##### Distribution.

Russia (south of Far East).

##### Etymology.

The specific name, *melanofuscella*, is derived from two Latin roots *melano*- and *fusc*-, collectively meaning “dark brown”, and refers to the forewing coloration of the new species.

#### Ypsolopha straminella sp. n.

urn:lsid:zoobank.org:act:47D44584-9A15-455C-A278-63D705EF79ED

http://species-id.net/wiki/Ypsolopha_straminella

Figs 6 , 12 , 14

##### Type material. 

**Holotype**: ♂, Russia, Primorskii krai, Khasanskii district, Mramornaya Mt. foot, 42°34'12"N, 130°48'30"E, 31 July –1 August 2003, M. Ponomarenko leg., gen. slide Yps.–62 MP (IBSS); labeled by red label with designation “Holotype”. **Paratypes** (41♂, 27♀): Russia. Amurskaya obl.: 2♂, 1♀, 17 km NNE Blagoveshchensk, altitude 160 m, 50°24'01"N, 127°40'20"E, 19, 20 August 2006, gen. slide Yps.–65 MP (♀); 2♂, 1♀, 15 km SSE Svobodnyi, Malaya Sazanka village vic., 51°14'03"N, 128°03'54"E, altitude 160 m, 21, 22 August 2006; 1♂, Mikhailovskii distr., 17 km NE Mikhailovka, Zavitaya riv., altitude 166 m, 50°023'23"N, 128°58'19"E, 15 August 2006, M. Ponomarenko leg. (IBSS). Khabarovskii krai: 1♀, Pivan’, 50°31'N, 137°04'E, 18 July 2007, V. Dubatolov, A. Syachina leg. (IBSS). Jewish Autonomous Region: 1♀, Smidovichiskii distr., Zabelovskii reserve, 8–14 July 2001, A. Streltcov, P. Osipov leg. (IBSS). Primorskii krai: 4♂, 1♀, same locality, collecting time and collector as in holotype, gen. slide Yps.–63 (♀) MP; 2♂, 3♀, same locality as in holotype, 2 August 2003; 2♂, 2 km NW Partizansk, Frentzovka village vic., altitude 400 m, 43°12'52"N, 133°01'27"E, 4–5 August 2002, gen. slide Yps.–64 MP; 13♂, 10♀, Ussuriyskii distr., Gornotaezhnoe, 43°41'42"N, 132°09'24"E, 20 July, 1 September, 1 October 1994; 17, 22, 23 August, 10, 21, 22, 23 September 1995, gen. slide Yps.–66 (♂) MP; 2♂, 42 km W Ussuriisk, 4 km N Monakino, 43°46'50"N, 131°26'29"E, altitude 290 m, 23 April 2003; 1♀, 5 km E Nikolo-Lvovskoe village, 43°52'16"N, 131°25'14"E, altitude 174 m, 2 September 2002, gen. slide Yps.–67 MP; 1♂, 1♀, 29 km SE Ussuriisk, 43°37'48"N, 132°13'44"E, 25 August 2001, M. Ponomarenko leg.; 1♂, 4,5 km SE Okeanskaya, 43°13'06"N, 132°03'26"E, 16 September 1993, E. Beljaev leg.; 1♀, Chernigovskii distr., 42 km S Spassk-Dalnii, Gribnoe village vic., 44°15'29"N, 132°44'51"E, 25 August 1998, gen. slide Yps.–68 MP; 5♂, 4♀, Khasanskii distr., 14 km SW Slavyanka, Ryazanovka, 42°47'36"N, 131°15'06"E, 9–10 September 1992; 23 August 1997; 19, 20, 21 September; 22 October 2008; 16 August 2010; 5♂, 2♀, Gamov Peninsula, Srednyaya Inlet, 42°35'18"N, 131°12'48"E, 24 July 1997, 8, 9, 13, 14 August 2009; 1♂, Gamov Peninsula, Gorshkova Inlet, 42°40'17"N, 131°12'48"E, 30 August 1997, M. Ponomarenko leg. (IBSS).

##### Diagnosis.

The new species resembles *Ypsolopha leuconotella* (Snellen, 1884) in the genitalia of both sexes: in the male in the shape of the valva with parallel margins in the distal half, the gnathos with a wide, rounded median plate, the almost straight aedeagus with indistinct cornuti of less than half the length of the aedeagus; and in the female in the presence of a scobinate zone in the ductus bursae. The new species differs from *leuconotella* in the male genitalia in the narrower base of the valva, which is less than one-half its greatest width (in *leuconotella* equal to one-half of greatest width), the rounded emargination between the anterior lobes of the tegumen (in *leuconotella* the emargination between the anterior lobes of the tegumen is triangular) and the inflated basal part of the aedeagus (caecum) (aedeagus narrowed basally in *leuconotella*). The new species can also be separated in the female genitalia by a scobinate zone in the ductus bursae of slightly over one-half the ductus length (in *leuconotella* the ductus with scobinate inner surface except 1/10 just following antrum), and a large signum, longer than two-thirds of corpus bursae (in *leuconotella* slightly less than one-half of corpus bursae length).

##### Description.

**Adult** (Fig. 6).Frons and vertex covered by closely fitting greyish yellow scales. Occiput with appressed elongated scales mainly greyish yellow and speckled with dark before light tips. Antenna filiform; scape greyish yellow with light yellow pecten; each flagellomere with light yellow basal and dark brown distal half; ventrally ciliated. Labial palpus porrect and slightly curved upwards, terminally pointed; basal segment white; second segment basally white, with triangular greyish yellow ventral tuft, speckled with brown; third segment as long as second, dorsally white, ventrally dark brown with scattered white scales. Thorax and tegula brownish grey. Foreleg with white femur and tibia; each tarsomere with narrow white distal ring, irrorated with brown. Midleg with white femur, tibia, ventral side of first (basal) tarsomere and spurs, second and following tarsomeres speckled with brown, lighter laterally and with narrow white ring distally. Hindleg with white femur, tibia, basal tarsomere and spurs; 2nd–5th tarsomeres speckled with brown and with white ventral margin. Forewing length 7.5–8.0 mm (n = 6), wingspan 16.5–17.3 mm (n = 4), sub-trapezoidal, with falcate, obtuse apex and sinuate termen. Ground colour of forewing greyish yellow irrorated with brown and greyish scales; 1/6th of costal margin brown; large greyish brown spot on dorsal half at 1/4, wide oblique brown band from base of R1 to middle of dorsal margin, large semi-oval greyish brown spot at end of cell, smaller concolorous spot dorsad and distad of latter, dorsal margin of wing and tornus greyish brown, six indistinct small strokes on costal margin from middle to 7/8; fringe greyish yellow. Hindwing dark brownish grey, darker towards apex; fringe brownish grey basally and yellow distally.

**Male genitalia** (Fig. 12). Uncus equal to vinculum in width, with almost straight posterior margin; socii 2.3 times length of median plate of gnathos, with long setae, curved outwardly before pointed apices. Median plate of gnathos with parallel lateral margins, one-third width of uncus. Tegumen divided into pair of anteriorly angulate lobes, anterior margin with triangular median emargination, not exceeding 1/4 of tegumen length. Valva more or less obovate, distally rounded and considerably narrowed towards base, with almost straight costal margin and rounded saccular margin; costal and saccular area equal in width, reaching 4/5 length of valva. Vinculum band–like, laterally with small triangular plates; saccus tapering towards apex, almost as long as socii. Anellus setose and wider than basal part of valva. Aedeagus almost straight, with cylindrical apex; coecum slightly longer than 1/4 length of aedeagus, slightly inflated in proximal part and wider than distal part of aedeagus; two long cornuti less than one-half length of aedeagus, each with distal needle–like part, less 1/5 total length of cornutus.

**Female genitalia** (Fig. 14). Papilla analis semi-oval, ovipositor telescopic, intersegmental membrane between IX and VIII almost four times length of VIII. Apophysis posterior slender, slightly thickened anteriorly, 2.75 times length of apophysis anterior; apophysis anterior with Y–shaped base, its ventral branch extending lateroventrally along anterior margin of VIII. Segment VIII, its ventral sclerotization, ostium and antrum very similar to *Ypsolopha melanofuscella* sp. n. Ventral sclerotization of VIII with sinuous anterior margin, in distal 1/3 divided into pair of rounded lobes, bearing long setae on rounded apex. Ostium slightly wider than one-half of segment VIII; antrum cone–shaped, separated from ductus bursae by narrowing and ring–like colliculum concave inward dorsally, its ventral part as narrow band and dorsal one 3 times wider. Ductus bursae tubular, membranous, 1.7 times length of corpus bursae, dilated anteriorly; anterior part with dense minute scobinations on inner surface from 2/5 to 1/14 length of ductus. Bulla seminalis shorter than corpus bursae, with ductus seminalis 1/3 length of bulla seminalis, arising from ductus bursae just near colliculum. Corpus bursae ovate, membranous; signum large band–like, comparatively wide and long, more than 2/3 length of corpus bursae, with two transverse ridges.

##### Distribution.

Russia (south of Far East).

##### Etymology.

The species name, *straminella*, is derived from the Latin *stramineus*, meaning “straw”, or “pale yellow” and refers to the colour of the forewing of the new species.

## Supplementary Material

XML Treatment for Ypsolopha asperella

XML Treatment for Ypsolopha costibasella

XML Treatment for Ypsolopha nigrofasciata

XML Treatment for Ypsolopha nigrimaculata

XML Treatment for Ypsolopha melanofuscella

XML Treatment for Ypsolopha straminella
